# Rising Leishmaniasis Cases in the United States Based on Registry Data From 2007 to 2023 and the Vital Role of Health Care Providers in Awareness and Management

**DOI:** 10.2196/65579

**Published:** 2025-06-20

**Authors:** Mia Panlilio, Olnita Martini, Elizabeth Tchernogorova, Alexa Carboni, Danielle Duffle, Leslie Torgerson

**Affiliations:** 1 Department of Biomedical Sciences, College of Osteopathic Medicine, Rocky Vista University, 8401 S Chambers Rd, Englewood, CO, 80112, United States, 1 303-373-2008; 2 College of Osteopathic Medicine, Rocky Vista University, Ivins, UT, United States

**Keywords:** leishmaniasis, leishmania, sandfly, awareness, management, health care provider, dermatology, dermatologist, skin condition, skin, dermis, United States, low-income population, low-income area

## Abstract

This letter highlights the increasing incidence of leishmaniasis cases in the United States, using the available data from Texas, and underscores the need for heightened awareness among health care providers regarding leishmaniasis diagnosis and prevention.

## Introduction

Leishmaniasis is a parasitic infection caused by the protozoa *Leishmania* via the female sandfly vector, including *Phlebotomus* and *Lutzmyia*, which are most prevalent in the tropics and subtropics [1]. Leishmaniasis infection can manifest in different forms, including localized cutaneous leishmaniasis, mucocutaneous leishmaniasis, and visceral leishmaniasis (known as *kala-azar*) [1,2]. Over 80 cases of endemic cutaneous leishmaniasis have been reported as endemic in the United States since 2017, specifically arising in Texas, Oklahoma, and Arizona [2]. Currently, leishmaniasis is only a reportable disease in Texas, meaning many cases across the United States may go unreported. Additionally, McIlwee et al [2] found that only 20% of cases were reported to the Texas Department of State Health Services (DSHS) between 2007 to 2023, despite such reporting being a legal requirement, potentially highlighting medical providers’ lack of awareness regarding human leishmaniasis. As environmental temperatures increase globally, the leishmaniasis vector and reservoir habitats have been expanding northward, potentially reaching southeastern Canada by 2050 [3]. By 2080, over 27 million North Americans could be at risk [3,4]. By using the available data from Texas, our aim is to acknowledge and highlight the potential risk of leishmaniasis cases in the United States, educate providers on the signs and symptoms, and encourage patient education on how to mitigate leishmaniasis spread.

## Methods

Leishmaniasis case data were collected from 2007 to 2023 through the Texas DSHS, which tracks leishmaniasis cases reported by providers in Texas [5]. Texas population data for 2007 to 2023 were sourced from the US Census Bureau, which collects data through surveys, censuses, and governmental administrative data. Incidence rates for leishmaniasis in Texas were then calculated for 2007 to 2023.

## Results

**Figure 1.**
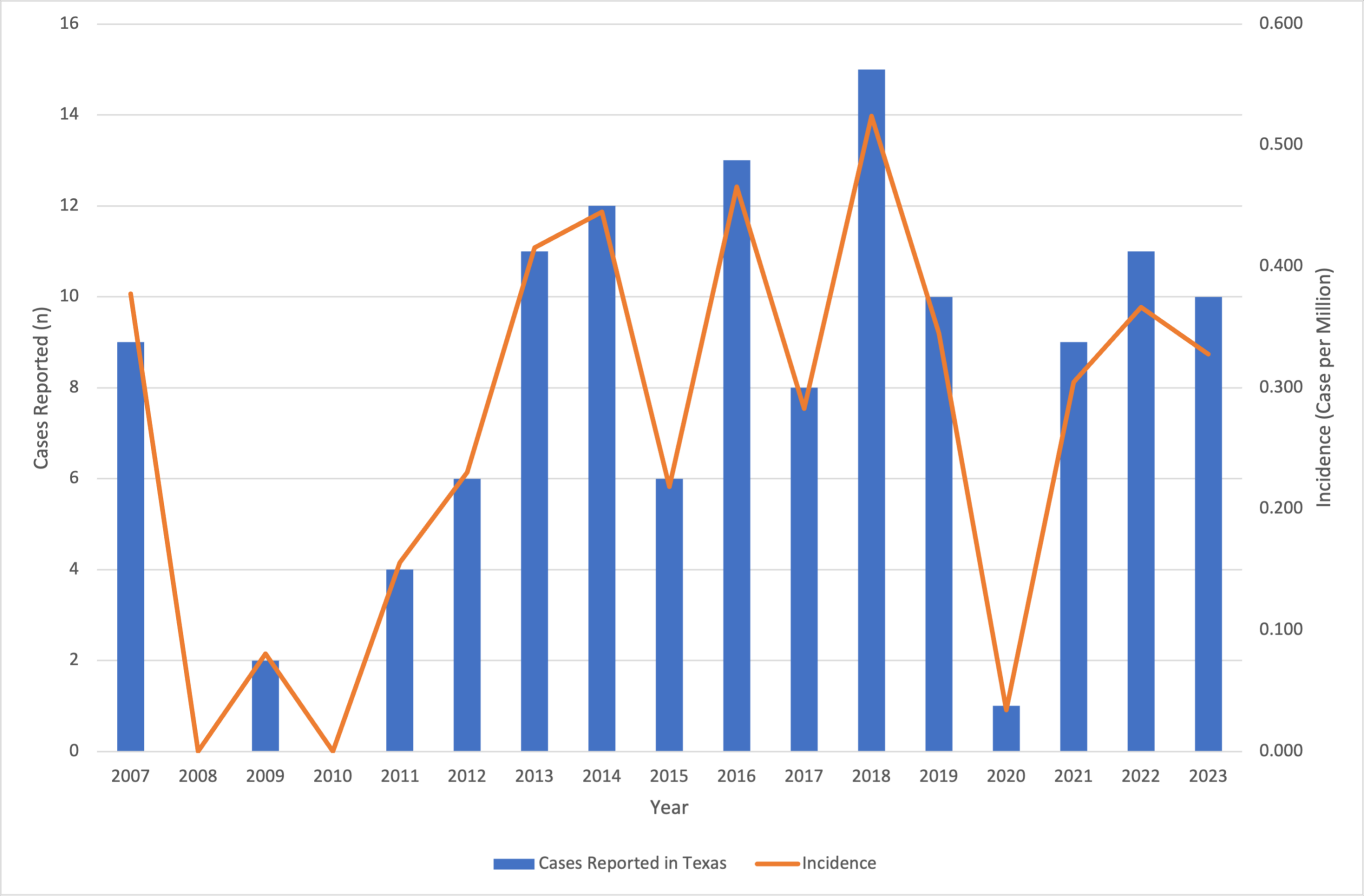
Leishmaniasis cases and incidence in Texas from 2007 to 2023. Annual reported leishmaniasis cases and incidence per million people in Texas are shown by the blue bars and orange line, respectively.

Between 2007 and 2023, the number of reported leishmaniasis cases in Texas fluctuated but trended upward over time, along with rising temperatures (Figures1  2). The number of cases rose from 9 in 2007 to a peak of 15 in 2018, with a slight decline afterward, reaching 9 again in 2023. Leishmaniasis incidence also increased from 0.378 per million in 2007 to 0.524 per million in 2018. A substantial drop to 0.304 per million occurred in 2020, with the 2023 incidence being slightly lower at 0.299 per million, indicating the disease’s ongoing presence.

**Figure 2.**
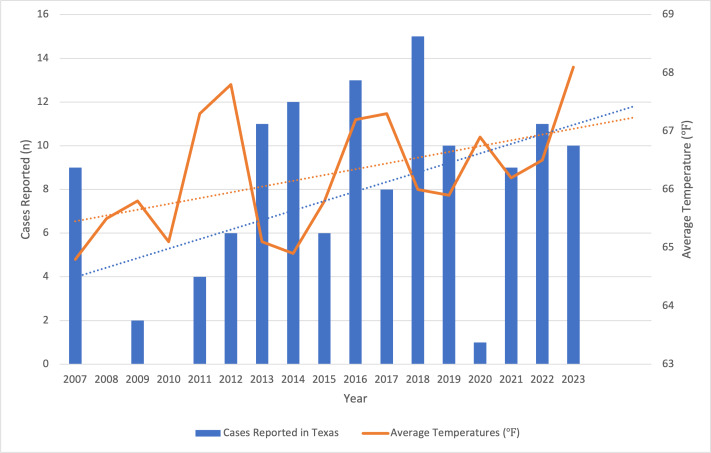
Annual reported leishmaniasis cases compared to the average temperature ([°F − 32][0.556] = °C) per year from 2007 to 2023.

## Discussion

### Principal Findings

The Texas data from 2007 to 2023 highlight a continual increasing trend in leishmaniasis incidence, which aligns with broader concerns regarding the emergence of this parasitic infection in the United States. The increase in reported cases—particularly seen from 2013 to 2018—may suggest improved awareness and reporting among health care providers. The fluctuations in recent years could be explained by the underreporting of cases (ie, only an estimated 20% of cases were reported to the Texas DSHS), which poses a significant epidemiologic issue.

The observed peaks in incidence, particularly in 2018, underscore the need for continued vigilance among health care providers in recognizing and diagnosing leishmaniasis. The drastic drop in incidence observed in 2020 is likely attributable to the COVID-19 pandemic, which altered exposure and travel patterns, reducing opportunities for leishmaniasis transmission. Additionally, disruptions to health care services and public health reporting during the pandemic may have contributed to the underreporting of cases. As the pandemic’s effects continue to influence public behavior, travel, and health care practices, it is probable that these factors are still impacting leishmaniasis incidence. The decrease in reported cases after 2018, along with the continued lower incidence observed in 2023, may reflect these ongoing effects and possible changes in environmental factors affecting the sandfly population.

This further emphasizes the necessity of nationwide reporting standards and greater education efforts among health care providers for ensuring early leishmaniasis detection and treatment. Diagnosis should include clinical assessment, travel history assessment, and laboratory tests such as skin biopsies and polymerase chain reaction assays. Early treatment is crucial for preventing complications, including topical antiparasitic medications for localized cases and systemic therapies for more severe involvement.

### Conclusion

This study’s findings should encourage public health officials and clinicians to have a high level of suspicion for leishmaniasis and prioritize surveillance and reporting, particularly in endemic regions like Texas. Mitigating continued spread can be addressed by patient education on preventative measures, such as covering exposed skin, wearing Environmental Protection Agency–registered insect repellent, and avoiding the outdoors after dusk. Medical personnel must be aware of important symptomatology to recognize leishmaniasis, including slowly ulcerating skin sores, swallowing difficulty, and nosebleeds. As numerous influences continue to increase leishmaniasis incidence, the United States must necessitate ongoing research and public health preparedness.

## Abbreviations

DSHS: Department of State Health Services
